# Progresses and Prospects on Glucosinolate Detection in Cruciferous Plants

**DOI:** 10.3390/foods13244141

**Published:** 2024-12-20

**Authors:** Xuaner Li, Dongna Wen, Yafei He, Yumei Liu, Fengqing Han, Jialin Su, Shangxiang Lai, Mu Zhuang, Fuxing Gao, Zhansheng Li

**Affiliations:** State Key Laboratory of Vegetable Biobreeding, Institute of Vegetables and Flowers, Chinese Academy of Agricultural Sciences, Beijing 100081, China; lixuaner057@163.com (X.L.);

**Keywords:** glucosinolate, cruciferous crops, HPLC, LC-MS/MS, determination

## Abstract

This review provides a comprehensive summary of the latest international research on detection methods for glucosinolates in cruciferous plants. This article examines various analytical techniques, including high-performance liquid chromatography (HPLC), liquid chromatography–mass spectrometry (LC-MS), enzyme-linked immunosorbent assay (ELISA), and capillary electrophoresis (CE), while highlighting their respective advantages and limitations. Additionally, this review delves into recent advancements in sample preparation, extraction, and quantification methods, offering valuable insights into the accurate and efficient determination of glucosinolate content across diverse plant materials. Furthermore, it underscores the critical importance of the standardization and validation of these methodologies to ensure reliable glucosinolate analyses in both scientific research and industrial applications.

## 1. Introduction

**Figure 1:**
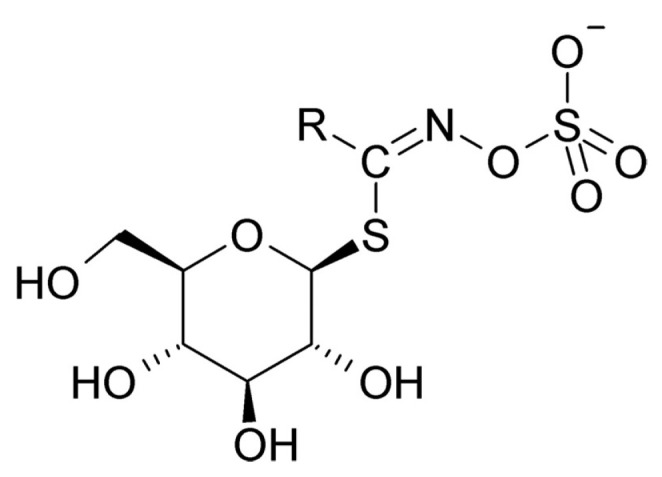
Molecular of glucosinolate structure.

Glucosinolates are a class of organic compounds found in cruciferous vegetables, such as broccoli, cabbage, kale, and Brussels sprouts [1,2,3]. These compounds have generated significant interest due to their potential health benefits and their role as bioactive compounds in plant defense mechanisms. The detection and quantification of glucosinolates are essential for both nutritional research and the advancement of plant breeding initiatives [4,5,6,7,8]. Glucosinolates are widely distributed across various plant parts, including roots, stems, leaves, and seeds. This widespread presence underscores the importance of their analysis. The significance of glucosinolates is not only attributed to their direct consumption by humans and animals but also to the potential health benefits they may confer [9,10]. The molecular structure of glucosinolates is illustrated in Figure 1 [11].

Currently, a variety of analytical techniques have been utilized to quantify glucosinolates in various plant tissues, including leaves, roots, buds, and seeds. The most frequently employed methods encompass HPLC, GC, and CE [12,13,14]. As previously mentioned, the content of glucosinolates can be analyzed either by directly measuring intact glucosinolates or by indirectly assessing their degradation products. However, certain analytical methods, such as gas chromatography (GC), may not be appropriate for direct analysis due to the non-volatile or thermally unstable properties exhibited by some glucosinolates. Consequently, it is often necessary to convert these analytes into volatile derivatives to facilitate effective detection. HPLC has been extensively utilized for the analysis of glucosinolates; however, the sample preparation process often necessitates desulfation. This procedure may lead to incomplete desulfation, self-dimerization, and self-degradation, which can potentially compromise the reliability of the final data. Furthermore, the absence of standards for certain desulfated compounds poses a significant challenge to this methodology [15].

Similar challenges arise when utilizing High-Resolution Mass Spectrometry (HRMS) for the detection of isomeric glucosinolates. This limitation can be mitigated by employing LC-MS/MS, which adds an additional dimension to mass spectrometric detection, allowing for the simultaneous scanning of precursor and fragment ions, thereby enhancing selectivity and sensitivity. Furthermore, compared with LC-MS, LC-MS/MS typically necessitates reduced analysis durations, facilitating the confirmation of known glucosinolates and the identification of novel compounds based on their fragmentation patterns [16,17]. Recently, using a novel computerized system, GLS Finder [18,19,20,21,22], the analysis of glucosinolates in 49 common cruciferous vegetables has been conducted using data generated from high-resolution HPLC-MS/MS techniques. However, some reviewers have pointed out that the reported results may be ambiguous, as the number of chromatographic peaks observed for glucosinolates or their isomers exceeds the known quantity of glucosinolates present in each extract [23]. This review aims to summarize the latest advancements in detection methods for glucosinolates in cruciferous plants, providing valuable insights for future research.

## 2. Determination Methods of Glucosinolates

**Figure 2:**
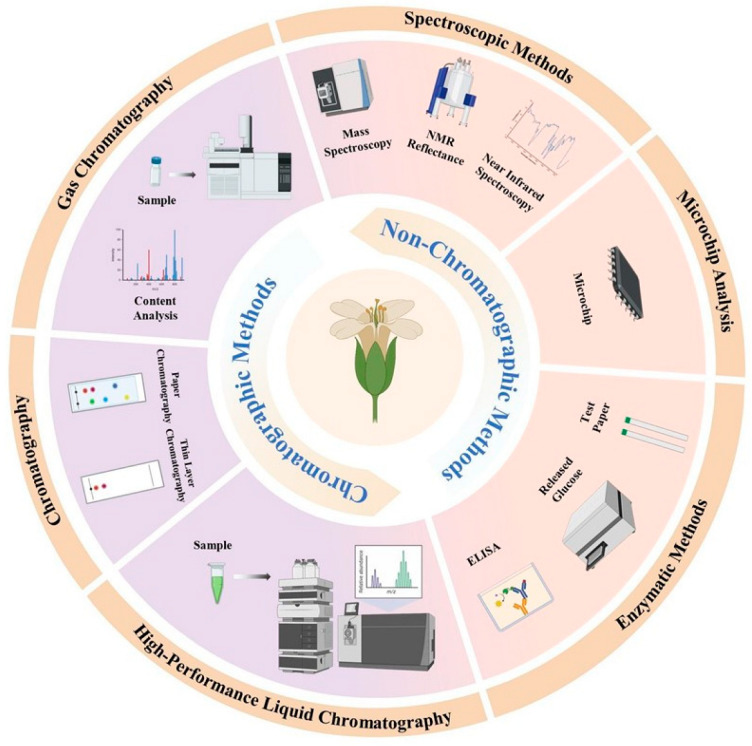
The characterization methods of glucosinolates.

Several methods have been developed for the detection of glucosinolates, with HPLC, LC-MS, GC-MS CE, ELISA, NIRS, and NMR being the most prominent (Figure 2). Each technique offers distinct advantages and limitations in terms of sensitivity, specificity, throughput, and cost. The choice of an appropriate method depends on specific analytical requirements such as the number of glucosinolate types to be detected, the desired level of quantification, and available resources. This section discusses these four detection technologies in detail.

### 2.1. HPLC

HPLC is a widely used general separation technique and one of the most extensively applied analytical technologies for glucosinolates. It is characterized by high sensitivity, good selectivity, and excellent reproducibility. The basis for HPLC to separate each chemical entity in a sample mixture relies on its unique affinity for the adsorbent material in the chromatographic column or mobile phase, resulting in various components moving at different rates and being separated. Previously known as high-pressure liquid chromatography, it relies on high-pressure pumps to accelerate the separation speed. HPLC separation primarily depends on inherent adjustable parameters of the mobile phase, such as polarity, flow rate, pH value, composition, and some intrinsic characteristics of the sample matrix; the type and nature of the stationary phase; and environmental factors like temperature and detector type and settings [24].

HPLC and reflectance spectroscopy each possess distinct advantages and applications in the analysis of glucosinolates. HPLC is recognized as the most reliable and precise technique, suitable for quantitative analysis in laboratory settings, providing detailed information on glucosinolate content. In contrast, reflectance spectroscopy serves as a non-destructive method that can rapidly assess the reflective characteristics of plant surfaces, indirectly indicating variations in glucosinolate concentrations within plants. This makes it particularly useful for rapid screening and large-scale monitoring. In practical applications, the combination of these two techniques can complement each other, enhancing both the efficiency and accuracy of glucosinolate analysis [25,26,27]. Lee et al. investigated the applicability of near-infrared reflectance spectroscopy (NIRS) for estimating glucosinolate content in whole seeds from various cruciferous plants. Their findings demonstrated the potential of reflectance spectroscopy to estimate glycosinolate concentrations in real-time without causing damage to crops while comparing it with high-performance liquid chromatography used for calibration [28]. Tian et al. employed liquid chromatography-electrospray ionization tandem mass spectrometry combined with selective reaction monitoring to detect glucosinolate levels effectively. This method successfully quantified ten different types of glucosinolates present in broccoli, broccoli sprouts, Brussels sprouts, and cauliflower while exhibiting higher selectivity and sensitivity towards these compounds [29].

In conclusion, HPLC stands as the conventional technique for glucosinolate analysis. Numerous novel methodologies have emerged that enhance the qualitative assessment of glucosinolates, thereby broadening their analytical applications.

### 2.2. LC-MS

LC-MS is a powerful technique that integrates the separation capabilities of liquid chromatography with mass spectrometric detection. It exhibits high sensitivity and specificity, enabling the simultaneous identification and quantification of multiple analytes. However, LC-MS is more complex and costly than HPLC, which limits its widespread application. The sample processing procedure closely resembles that of HPLC, involving the extraction of thioglucosides using appropriate solvents followed by separation via liquid chromatography upon completion. The separated components are then introduced into the mass spectrometer, which analyzes ions based on their mass-to-charge ratio (*m*/*z*) to provide molecular mass and structural information. Key advantages include enhanced sensitivity and selectivity, suitability for complex samples, and the capacity to yield insights into molecular structure [30,31,32,33]. Mass spectrometry (MS), owing to its high sensitivity, has been employed for the identification and quantification of glucosinolate compounds. A method has also been developed that integrates liquid chromatography with MS without necessitating desulfation. However, the accuracy and precision of this method are constrained by insufficient mass resolution. For instance, the mass-to-charge ratios (*m*/*z*) of glycosides and glycoproteins (*m*/*z* 422.0255 and *m*/*z* 422.0585, respectively) are remarkably similar. Although they share the same nominal mass of 422, their isotopic masses differ by only 0.033 Da, making it challenging to distinguish them through mass analysis alone. While these compounds can be separated chromatographically, the application of LC-MS remains limited if certain glucosinolate peaks significantly overlap or if there is substantial background interference in the monitoring channel [34].

Recent studies have demonstrated the effectiveness of LC-MS in identifying various glucosinolates. For instance, Szűcs et al. reported the detection of several minor glucosinolates, including glucoraphanin and glucoiberin, in horseradish roots using liquid chromatography–electrospray ionization mass spectrometry (LC-ESI-MS) [35]. Similarly, Klimek-Szczykutowicz et al. utilized LC-MS/MS to identify five glucosinolates in methanol extracts of cress, further underscoring the versatility of LC-MS in analyzing complex plant matrices [36]. Given the structural diversity of glucosinolates, this capability is particularly significant as substantial variations may exist even within a single plant species. The application of LC-MS extends beyond identification; it also facilitates the quantification of glucosinolates. For example, Xu et al. developed a hydrophilic interaction liquid chromatography–tandem mass spectrometry (HILIC-MS/MS) method capable of simultaneously quantifying 22 different glucosinolates from various cruciferous vegetables, showcasing both efficiency and sensitivity [37]. Furthermore, the integration of tandem mass spectrometry (MS/MS) allows for the elucidation of glucosinolate structures through fragmentation patterns that can be compared with known standards [38]. This approach not only aids in confirming the identity of glucosinolates but also enhances the reliability of quantitative analyses. Moreover, advancements in LC-MS methodologies have improved detection limits and enabled the analysis of glucosinolates within complex biological samples. For instance, Hauder et al. developed a sensitive LC-MS/MS method for quantifying metabolites derived from glucosinolates in human plasma and urine, highlighting its applicability in nutritional research [39]. This is crucial for understanding the bioavailability and metabolism of dietary-derived glucosinolates post-consumption—factors that significantly impact human health and disease prevention. In summary, LC-MS has become an indispensable tool for analyzing glucosinolates by providing comprehensive insights into their identification, quantification, and metabolic pathways. Ongoing improvements to LC-MS technology are expected to enhance our understanding of gluconate chemistry and its implications for plant biology as well as human health.

### 2.3. GC-MS

Gas chromatography–mass spectrometry (GC-MS) is a commonly utilized analytical technique for the analysis of thioglucosides and their decomposition products (such as isothiocyanates) [40,41,42]. GC-MS possesses high separation efficiency and sensitivity, enabling the detection and identification of various thioglucosides [43,44]. The GC-MS analysis of complete glucosinolates typically encompasses sample preparation steps, such as extraction and derivatization [37,39]. Glucosinolates are usually transformed into desulfurized forms or volatile decomposition products, like isothiocyanates, prior to GC-MS analysis [23,45]. This step is requisite because complete glucosinolates are insufficiently volatile to undergo direct GC-MS analysis [46,47]. Numerous studies have reported the identification and quantification of glucosinolates in various plant species (including cruciferous vegetables, Lepidium species, and Moringa oleifera) using GC-MS [41,42,48,49]. GC-MS analysis provides detailed information on the glucosinolate profile, including the identification of individual glucosinolate compounds and their relative contents.

### 2.4. ELISA

ELISA is a fast, cost-efficient, and simple technique for detecting glucosinolates. It relies on the specific interaction between antibodies and glucosinolate molecules. ELISA is particularly effective for high-throughput screening, although it may suffer from issues such as cross-reactivity and limited specificity. The method uses antibodies that are specifically designed to target the glucosinolate of interest, binding to the molecule and generating a detectable signal via an enzymatic reaction, making it highly suitable for the quantitative measurement of particular glucosinolates [50,51]. Before analysis, it is necessary to prepare specific antibodies for the target glucosinolate and then process the sample. After the antibodies are ready, the extracted sample is diluted and added to the ELISA plate, where antigen–antibody binding occurs. A substrate is then introduced, and the enzyme reacts with it, resulting in a color change that can be measured with a spectrophotometer. While ELISA offers high sensitivity and specificity, making it ideal for large-scale screening, it has certain drawbacks, including the need for prior antibody preparation, a more complex procedure, and limited ability to analyze unknown compounds.

The exploration of glucosinolates quantification has been conducted using ELISA in conjunction with other analytical methods such as HPLC and mass spectrometry. For instance, studies have demonstrated that HPLC can effectively separate and quantify various types of glucosinolates, including aliphatic and indole glucosinolates, which is crucial for understanding their biological activities and potential health benefits [33,52]. However, ELISA offers a simpler and faster alternative for screening large numbers of samples, which is particularly beneficial for breeding programs aimed at developing crops with enhanced glucosinolate profiles [53]. In the context of Brassica species, the profile of glucosinolates can vary significantly based on genetic and environmental factors. Research indicates that different varieties of cabbage exhibit distinct glucosinolate compositions, potentially linked to the expression of specific biosynthetic genes [52,54]. The integration of genetic studies with ELISA can facilitate the identification of key genes involved in glucosinolate biosynthesis, thereby aiding in the selection of varieties with optimal levels of these compounds for agricultural and nutritional purposes [55,56]. Optimizing sample extraction and preparation processes for enzyme-linked immunosorbent assays (ELISAs) enhances the recovery rate of glucosinolates. Techniques such as desulfurization and enzymatic hydrolysis are commonly employed to convert glucosinolates into detectable forms suitable for ELISA analysis [57,58]. Developing reliable extraction protocols is critical to ensuring accurate quantification since the content of glucosinolates can be influenced by factors such as plant maturity, processing methods, and storage conditions [59].

In summary, incorporating ELISA into research on mustard oil glycosides provides a valuable method for rapid and effective quantification of these compounds within plant tissues. By leveraging both ELISA’s capabilities alongside traditional methods like HPLC, researchers can gain deeper insights into the genetic and environmental factors affecting glucosinolate profiles—ultimately contributing to the development of crops with enhanced health benefits.

### 2.5. CE

Thiocyanates are bioactive compounds widely found in various plants, particularly within the Brassicaceae family. Capillary electrophoresis (CE) has emerged as a powerful analytical technique for the separation and analysis of thiocyanates. This method offers distinct advantages due to its high resolution, low sample volume requirements, and compatibility with complex biological matrices. Gonda et al. highlighted the role of CE in analyzing the thiocyanate–myrosinase–isothiocyanate system, emphasizing its ability to tolerate proteins and other macromolecules—an essential feature for analyzing plant extracts containing thiocyanates alongside various other components [60].

The use of CE for separating thiocyanates presents challenges, especially when dealing with real plant matrices. However, the inherent capabilities of this technology allow the effective separation of ionized compounds based on charge-to-size ratios, which is beneficial for thiocyanates possessing different ionic characteristics [60]. The integration of mass spectrometry (MS) with CE further enhances the sensitivity and specificity of thiocyanate analysis, enabling the identification and quantification of individual components within complex mixtures [61]. The combination of CE and MS has been shown to provide high-resolution profiles of polysaccharide structures; similarly intricate analyses can be applied to thiocyanates [62]. Moreover, advancements in capillary coating technologies and developments in micellar electrokinetic chromatography (MEKC) have improved the separation efficiency of thioglycosides within CE systems. These innovations facilitate better interactions with analytes while enhancing both resolution and sensitivity [60,63]. For instance, Zhang et al. discussed the application of polyvinyl alcohol-coated capillaries that significantly enhance stability and sensitivity during CE analysis—making it suitable for examining highly polar molecules such as thioglycosides [63].

In conclusion, capillary electrophoresis represents a robust and versatile method for analyzing thioglucosides characterized by high sensitivity, low sample consumption, and an ability to resolve complex mixtures. When combined with advanced detection methods like mass spectrometry, its applicability to phytochemical analysis is further augmented—rendering it a valuable tool for investigating the health benefits and biochemical roles associated with thiosugars present in diverse plant species.

### 2.6. NIRS

Near-infrared spectroscopy (NIRS) is a well-established technique for the rapid, non-destructive, and simultaneous analysis of various seed quality parameters in Brassica species, including glucosinolates. Several studies have demonstrated the effectiveness of NIRS for quantifying glucosinolates in Brassica seeds and leaves. For example, Ratajczak et al. Ratajczak et al. used NIRS calibrations to analyze seed components such as total glucosinolates, alkenyl glucosinolates, and individual glucosinolates like gluconapin, glucobrassiconapin, progoitrin, napoleiferin, and glucobrassicin in oilseed rape [64]. Similarly, Barthet et al. and Kumar et al. showed that the optimal spectral regions for predicting total glucosinolates in canola and rapeseed–mustard seeds are in the near-infrared range [65,66]. Jasinski et al. and Niemann et al. further highlighted the utility of NIRS for high-throughput phenotyping of various seed quality traits, including glucosinolates, in Arabidopsis and Brassica species [67,68]. They demonstrated that NIRS is a powerful, non-destructive method to assess the content of major seed components, such as oil, protein, and glucosinolates, and can be used to analyze natural variation in these traits. Additionally, several studies have explored the use of NIRS, in combination with chemometric techniques like partial least squares regression (PLSR) and stepwise multiple linear regression (SMLR), for the quantification of glucosinolates and other bioactive compounds in Brassica leaves and other plant materials [64,69,70,71,72].

Overall, the reviewed references provide strong evidence that NIRS is a reliable and efficient technique for the analysis of glucosinolates and other quality parameters in Brassica species, offering advantages such as speed, non-destructive analysis, and the ability to simultaneously quantify multiple components [65,73,74,75,76,77,78,79].

### 2.7. NMR

Nuclear Magnetic Resonance Spectroscopy (NMR) has been widely used for the structural elucidation and characterization of complete thioglucosides. Some studies have utilized 1H, 13C, and 2D NMR techniques to identify and differentiate various thioglucoside structures [80]. 1H and 13C NMR spectra provide characteristic signals for the thioglucose moiety, sulfate imido group, and side chains, allowing for the unambiguous identification of individual thioglucosides [80]. For example, Badenes-Perez et al. confirmed the identity of 3-methoxybenzyl sinigrin (glucoside) in Limnanthes douglasii based on NMR analysis of the complete sinigrin. The data were similar to previously reported NMR data for the same compound. Furthermore, they determined that 3-hydroxybenzyl thioglucoside is another thioglucoside present in the plant. Jaafaru et al. used 1H, 13C, HSQC, and COSY NMR analysis to differentiate between the rhamnose and glucose fractions in the sulfoglucoside-rich extracts of moringa and to identify the presence of aromatic rings, especially the characteristic phenyl group of sulfoglucosides [81]. Furthermore, Cannavacciuolo et al. reported the clear characterization of a new hydroxycinnamic acid derivative 1-O-feruloyl-2-O-sinapoyl-β-D-glucopyranoside in radish sprouts (Raphanus sativus) using NMR analysis [82]. They also identified glucosinolate benzoyl glucosinolate in the same plant material. The reference also emphasizes the combination of NMR spectroscopy with other analytical techniques such as HPLC and mass spectrometry to provide a comprehensive profile of thioglucoside spectra in various plant species [83,84,85].

In summary, the provided references demonstrate the valuable role of NMR spectroscopy in structural elucidation and identification of complete thioglucosides. The combination of 1H, 13C, and 2D NMR techniques can clearly characterize the thioglucose moiety, sulfate imido group, and variable side chains, enabling the identification of individual thioglucoside structures in complex plant extracts.

## 3. Qualitative and Quantitative of Glucosinolates

### 3.1. Sample Preparation

The preparation of samples for glucosinolate analysis is a critical step that significantly impacts the integrity and quantification of these compounds. Various methods and conditions have been investigated to optimize glucosinolate extraction, each carrying distinct implications for the final results. A key concern in the preparation of glucosinolate samples is the potential degradation of these compounds during processing. For example, González-Hidalgo emphasized that the time elapsed between harvesting and processing can lead to cellular damage, facilitating contact between glucosinolates and myrosinase, which results in rapid hydrolysis and a subsequent reduction in glucosinolate concentrations compared with fresh samples [86]. This degradation highlights the necessity of minimizing the interval between harvest and processing to preserve glucosinolate levels.

Furthermore, the application of lyophilization (freeze-drying) may inadvertently diminish glucosinolate levels, as noted by Major et al., who observed that various drying methods did not significantly affect glucosinolate content in certain instances, indicating that careful consideration of drying techniques is crucial [87]. The extraction process can also be affected by the physical state of the plant material. For instance, mechanical processing of vegetables, such as shredding, may initially reduce glucosinolate levels but could subsequently result in an accumulation of these compounds over time due to the release of myrosinase [88]. This phenomenon suggests that while immediate processing may result in losses, subsequent reactions can potentially enhance glucosinolate levels, underscoring the necessity for a balanced approach in sample preparation. Furthermore, the analytical methods utilized for glucosinolate quantification must be both robust and reliable. Techniques such as HPLC are frequently employed; however, the extraction and purification steps can be labor-intensive and time-consuming [89]. Grosser and Dam propose a streamlined HPLC method that simplifies the extraction process while ensuring accuracy [90]. This is critical as the complexity of glucosinolate profiles necessitates precise analytical techniques to ensure accurate quantification.

In conclusion, the preparation of samples for glucosinolate analysis necessitates meticulous attention to detail, encompassing the timing of processing, selection of extraction solvents, and employed methodologies. The interplay among these factors can significantly influence the final glucosinolate content and its bioactive potential. Therefore, adopting optimized extraction protocols and analytical methods is imperative for accurate glucosinolate profiling.

### 3.2. Extraction

Several extraction methods have been utilized to isolate glucosinolates from plant materials, including solvent extraction, ultrasound-assisted extraction (UAE), and microwave-assisted extraction (MAE). The selection of the optimal extraction method is critical for achieving high recovery rates and purity of glucosinolates. The effective extraction of glucosinolates—bioactive compounds present in cruciferous vegetables—can be accomplished through various techniques, each possessing unique advantages and challenges that are essential for optimizing extraction efficiency. Solvent extraction remains a conventional method for isolating glucosinolates, where the choice of solvent significantly affects the yield and purity of the extracted compounds. For instance, studies have demonstrated that an 80% methanol solution can effectively inactivate myrosinase, an enzyme responsible for degrading glucosinolates, thereby preserving their concentrations during extraction [91,92]. Furthermore, the selection of extraction solvent and method is crucial for optimizing glucosinolate recovery efficiency. Cold methanol extraction has demonstrated effectiveness, as it not only preserves glucosinolate content but also mitigates complications associated with elevated temperatures that may lead to degradation [91]. Doheny-Adams et al. demonstrate that cold methanol extraction surpasses other methods, particularly in preserving glucosinolate concentrations, thereby establishing it as a preferred technique for sample preparation [91]. Furthermore, the application of hydroalcoholic mixtures has been shown to enhance glucosinolate extraction yields, with varying efficiencies observed depending on solvent composition [93]. However, traditional solvent extraction often necessitates substantial time and solvent volumes, which may result in increased operational costs and environmental concerns [94].

In contrast, UAE has emerged as a more efficient alternative. This method employs ultrasonic waves to generate cavitation bubbles that disrupt plant cell walls and facilitate the release of glucosinolates into the solvent [95,96]. For instance, studies have demonstrated that UAE achieves higher extraction efficiencies for glucosinolates from Camelina sativa by optimizing parameters such as solvent type and extraction duration [96]. Additionally, the application of UAE has been shown to improve the extraction of phenolic compounds, which are often co-extracted with glucosinolates, thereby further increasing the nutritional value of the extracts [97]. MAE is a technique that has garnered attention for its capacity to enhance extraction efficiency. MAE utilizes microwave energy to heat both the solvent and sample, resulting in the rapid extraction of glucosinolates due to the elevated temperature and pressure within the plant matrix [98,99]. Studies have demonstrated that MAE can yield results comparable to or superior to those obtained through UAE and traditional solvent extraction methods, particularly regarding extraction speed and solvent efficiency [94,98]. The integration of MAE with optimization techniques such as response surface methodology has been shown to maximize glucosinolate yields while minimizing solvent consumption [99]. In summary, while traditional solvent extraction methods are effective for glucosinolate extraction, UAE and MAE present significant advantages in terms of efficiency, yield, and environmental impact. The selection of the appropriate extraction method should be informed by the specific requirements of the target glucosinolates and the desired purity of the final extract.

### 3.3. Purification

An effective method for extracting thiocyanates is HPLC, which can isolate and quantify these compounds from plant tissues. Grosser and Dam described a straightforward approach using HPLC for the extraction and analysis of thiocyanates, emphasizing that their technique can be applied to samples with low concentrations of thiocyanates, such as soil [90]. This method does not require freeze-drying; due to the moisture content in fresh plant materials, freeze-drying complicates the quantification process. Similarly, Doheny-Adams demonstrated that cold methanol extraction is highly effective for isolating thiocyanates, outperforming other methods except for specific compounds like rapeseed oil derivatives [91]. Their findings underscore the importance of utilizing appropriate solvents and conditions to maximize thiocyanate yield. The purification process typically involves chromatographic techniques. For instance, Saha et al. reported on obtaining glucosinolate through glucose mustard oxidation followed by purification using DEAE-Sephadex and Sephadex G10 [100]. This highlights the practicality of ion-exchange chromatography in separating specific thiocyanates. Additionally, Hebert et al. investigated the use of macroporous anion exchange resins for separating and purifying mustard oil glucosinolates while optimizing processes to enhance recovery rates [101]. Their work emphasizes the versatility of chromatographic methods in purifying thiocyanates.

Furthermore, ultrasonic-assisted extraction has been studied as a means to increase yields of thiocyanates. Martínez-Zamora et al. combined ultrasound with post-harvest processing to improve synthesis efficiency for sulforaphane—a notable type of thiocyanate—demonstrating that this method not only aids in extraction but also potentially enhances the biological activity of extracted compounds [102].

In addition to these methodologies, desulfurization’s role in purifying thiocyanates has been highlighted by Chengzhi et al. and Xie et al. They noted that desulfurization is a critical step in preparing analyzable forms of thiocyantes since it converts them into more suitable formats for detection and quantification via HPLC. This step is essential for accurately assessing various plant extracts’ profiles concerning their thiosulfinate content [103,104]. In conclusion, purifying thiosulfinate represents a multifaceted process integrating diverse extraction and chromatographic techniques. The choice of methodology significantly impacts both the yield and purity of final products; thus, selecting an appropriate strategy based on target-specific thiosulfinate types and source materials is crucial (Table 1).

### 3.4. Quantification

The quantification of glucosinolates can be accomplished by constructing calibration curves with standard compounds or by comparing the peak areas of samples against those of internal standards. The choice of a quantification method is dependent on the analytical technique utilized and the availability of suitable standards. The quantification of glucosinolates, a class of sulfur-containing compounds predominantly present in the Brassicaceae family, is essential for elucidating their biological activities and nutritional significance. Various methodologies have been developed to accurately assess glucosinolate content in plant tissues, each possessing distinct advantages and limitations.

HPLC is one of the most widely employed techniques for quantifying glucosinolates. This method facilitates the separation and identification of individual glucosinolates based on their distinct chemical properties (Table 2). For instance, research has demonstrated that HPLC can effectively differentiate among various glucosinolates present in different cruciferous crops and Brassica species, thereby providing comprehensive profiles of their content [40,105,106]. The quantification process generally entails the extraction of glucosinolates from plant tissues, followed by hydrolysis to liberate glucose, which is subsequently measured spectrophotometrically [65,107]. This methodology has been validated in numerous studies, affirming its reliability for both total and individual glucosinolate measurements (Table 3) [99,108,109,110].

Concurrently, spectrophotometric methods have been developed for the rapid and cost-effective quantification of glucosinolates. For instance, a straightforward spectrophotometric approach was proposed to estimate total glucosinolates in mustard de-oiled cake, illustrating the potential for less resource-intensive techniques [116]. Furthermore, near-infrared spectroscopy (NIRS) has emerged as a promising alternative for non-destructive analysis of glucosinolates in intact seeds and plant tissues [53,117]. This method capitalizes on the unique spectral signatures of glucosinolates, enabling swift assessments without extensive sample preparation.

The quantification of glucosinolates is influenced by a multitude of factors, including plant species, developmental stages, and environmental conditions. For example, the content of glucosinolates can vary significantly among different cultivars of Brassica and in response to abiotic stresses such as salinity and temperature [75,99,118,119]. Furthermore, genetic factors play a crucial role in determining the glucosinolate profiles of different plant varieties. This knowledge can be utilized in breeding programs designed to enhance nutritional quality [55,120,121]. Additionally, the biological activity of glucosinolates is closely associated with their hydrolysis products—such as isothiocyanates—which are well-known for their health-promoting properties [99,122,123]. Therefore, understanding quantification methods not only facilitates the assessment of the nutritional value of Brassica vegetables but also provides insights into their potential health benefits. The quantification of glucosinolates is a complex process that can be accomplished using various analytical techniques, primarily HPLC and spectrophotometry. The selection of a method may be influenced by particular research objectives, the resources at hand, and the nature of the samples being analyzed. Further research into the factors affecting glucosinolate content will enhance our understanding of these important phytochemicals.

## 4. Standardization and Validation of Glucosinolate Detection Methods

### 4.1. Standardization

Standardizing glucosinolate detection methods is crucial to ensure accurate and reliable results. This involves establishing standardized protocols for sample preparation, extraction, and quantification, as well as using certified reference materials and participating in inter-laboratory comparison studies. The standardization of these methodologies is crucial for ensuring consistency and reliability in the analysis of bioactive compounds present in cruciferous vegetables, which provide significant health benefits. Various methodologies have been developed and validated, each with its own advantages and limitations.

HPLC is a widely employed technique for the analysis of glucosinolates. Vastenhout et al. have developed a method to evaluate the kinetics of glucosinolate hydrolysis using HPLC, demonstrating that absorbance exhibits a linear relationship with concentration, as confirmed by UV-Vis spectroscopy. This relationship is crucial for ensuring accurate quantification [124]. Similarly, Gallaher et al. developed and validated a spectrophotometric method for quantifying total glucosinolates in cruciferous vegetables, highlighting the reliability and speed of their approach, which has broad applicability [54]. The use of HPLC in combination with MS has also been emphasized by Frank et al., who employed LC-TOF-MS to screen mustard seeds for glucosinolates, demonstrating the method’s effectiveness in the identification and quantification of these compounds [42]. Additionally, Nuclear Magnetic Resonance (NMR) spectroscopy has been proposed as a promising method for the quantification of glucosinolates. Yuan et al. observed that although NMR is not commonly utilized for this purpose, it offers advantages such as eliminating the need for calibration standards, which can streamline the analysis process [40]. This is particularly pertinent when considering the complexity of plant matrices, where conventional methods may encounter difficulties due to interference from other compounds.

The extraction protocols employed prior to analysis are equally critical and can significantly influence the results. Neal et al. presented a methodology for the extraction of glucosinolates from leaves of Arabidopsis thaliana, wherein the identities of the peaks were validated using standards, thereby ensuring the reliability and accuracy of the findings [125]. Furthermore, Major et al. examined the influence of myrosinase activity during the extraction process, highlighting that enzymatic activity can result in a decrease in total glucosinolate concentration. This underscores the necessity for meticulous control over extraction conditions [87]. Recent advancements have also concentrated on optimizing extraction methods to enhance glucosinolate yield. For instance, Meza et al. developed a UPLC-DAD method that refined sample preparation procedures to achieve high specificity and accuracy in glucosinolate quantification [92]. Furthermore, ultrasound-assisted extraction has been investigated as a more environmentally friendly alternative, demonstrating the potential to improve extraction efficiency while minimizing solvent usage [96]. Thus, the standardization of glucosinolate detection methods necessitates a combination of reliable analytical techniques, optimized extraction protocols, and careful consideration of enzymatic activity. The integration of HPLC with MS, along with emerging methodologies such as NMR and ultrasound-assisted extraction, constitutes a comprehensive approach to glucosinolate analysis that can enhance the accuracy and reproducibility of results across various studies.

### 4.2. Validation

The validation of glucosinolate detection methods involves evaluating key parameters such as linearity, precision, accuracy, limit of detection (LOD), and limit of quantification (LOQ). This rigorous process ensures that the method is suitable for its intended application and generates reliable results. The validation of these methods is crucial to affirm their dependability and appropriateness for specific uses. Essential parameters for validation include linearity, precision, accuracy, LOD, and LOQ; each parameter plays a vital role in confirming that the analytical methods utilized can consistently deliver valid results. Linearity refers to the method’s capacity to yield results that are directly proportional to the analyte concentration within a specified range. For instance, studies have shown that HPLC methods can achieve exceptional linearity for glucosinolate quantification, with correlation coefficients (R^2^) frequently exceeding 0.99, thereby indicating a robust linear relationship between concentration and response [45,90]. This is crucial for ensuring that the method can accurately quantify glucosinolates across a spectrum of concentrations typically encountered in plant samples.

Precision, which evaluates the reproducibility of the method, is another critical parameter for validation. It is typically assessed through repeatability and intermediate precision studies. For instance, the precision of HPLC methods for glucosinolate analysis has been reported with relative standard deviations (RSDs) generally below 5%, indicating high reproducibility in measurements [90,91]. Similarly, methodologies such as UPLC-DAD have demonstrated consistent results across various laboratories, further reinforcing the reliability of these techniques [92,99]. Accuracy, which quantifies the proximity of measured values to true values, is a fundamental aspect of method validation. Various studies have utilized recovery experiments to evaluate accuracy, wherein known quantities of glucosinolates are added to samples, and the recovery rates are subsequently calculated. Reports indicate that recovery rates for glucosinolates typically range from 90% to 110%, thereby confirming the reliability of the methods employed [45,91]. Such accuracy is essential for ensuring that reported glucosinolate levels accurately reflect true concentrations in the samples.

LOD and LOQ are essential parameters for evaluating the sensitivity of analytical methods. LOD, or limit of detection, refers to the lowest concentration of an analyte that can be reliably identified, whereas LOQ, or limit of quantification, signifies the minimum concentration that can be quantified with acceptable precision and accuracy. Studies have demonstrated that HPLC methods can achieve LODs in the low micromolar range for various glucosinolates, rendering them suitable for detecting trace levels of these compounds in complex matrices [45,90]. This sensitivity is particularly vital in food safety and nutritional studies, where low glucosinolate levels may carry significant biological implications. The validation of glucosinolate detection methods through an assessment of linearity, precision, accuracy, LOD, and LOQ is essential to ensure result reliability. A rigorous evaluation of these parameters confirms that the employed methods are appropriate for their intended purposes, thereby bolstering research and applications across fields such as nutrition, food science, and plant biology.

## 5. Future Prospects

### 5.1. Development of New Analytical Techniques

The development of novel analytical techniques, such as ion mobility spectrometry (IMS) and NMR, has the potential to significantly enhance the sensitivity, selectivity, and throughput of glucosinolate analysis. Glucosinolates, which are sulfur-containing compounds present in cruciferous vegetables, have garnered significant attention due to their health benefits and roles in plant defense mechanisms. Traditional methods for glucosinolate analysis—primarily HPLC—while effective, may be limited in terms of sensitivity and specificity. The integration of advanced analytical techniques like IMS and NMR can effectively address these limitations.

NMR spectroscopy is particularly valuable due to its non-destructive and highly informative characteristics. Recent advancements in NMR technology, including the development of hyperpolarization techniques, have significantly enhanced the sensitivity of NMR measurements. For instance, hyperpolarized water has been employed to improve the detection of various compounds, including glucosinolates, by increasing the signal-to-noise ratio in NMR experiments [126,127]. Furthermore, the application of dynamic nuclear polarization (DNP) has demonstrated promise in further enhancing NMR sensitivity, facilitating the detection of low-abundance metabolites—crucial for analyzing glucosinolates within complex biological matrices [128]. Furthermore, the incorporation of magnetic nanoparticles in conjunction with NMR has emerged as a potent strategy for enhancing detection capabilities. Magnetic nanoparticles can alter the local magnetic environment, thereby influencing the relaxation rates of adjacent nuclei and improving the overall sensitivity of NMR measurements [129,130]. This approach is particularly advantageous for glucosinolate analysis, as it facilitates the detection of these compounds at lower concentrations and within more complex samples.

IMS is another promising technique that can complement NMR in the analysis of glucosinolates. IMS facilitates rapid separation and identification of ions based on their mobility in a gas phase, thereby providing high-throughput analytical capabilities. The integration of IMS with MS further enhances the specificity of glucosinolate detection by enabling the identification of specific molecular ions associated with glucosinolates and their degradation products [131,132]. This dual approach significantly improves the analytical workflow, allowing researchers to obtain comprehensive profiles of glucosinolates across various samples. The incorporation of sophisticated analytical methodologies, including NMR and IMS, offers a significant opportunity to enhance the analysis of glucosinolates. These methods not only improve sensitivity and selectivity but also facilitate high-throughput analysis, rendering them invaluable tools in the investigation of these important phytochemicals.

### 5.2. Miniaturization and Automation

The adoption of advanced analytical techniques, including NMR and IMS, provides a considerable opportunity to enhance the analysis of glucosinolates. These methodologies not only improve sensitivity and selectivity but also facilitate high-throughput analysis, thereby establishing them as essential tools in the investigation of these important phytochemicals. HPLC and Ultra-Performance Liquid Chromatography (UPLC) represent the cutting edge of glucosinolate analysis. Notably, UPLC is recognized for its superior separation efficiency, reduced solvent consumption, and shorter run times compared with conventional HPLC methods. For example, Meza et al. demonstrated that UPLC provides a more environmentally sustainable quantification of glucosinolates in Camelina seeds, thereby minimizing ecological impact while enhancing throughput [92]. Similarly, Shi et al. underscored the efficacy of HPLC coupled with diode array detection for quantifying glucosinolates, highlighting its significance in high-throughput applications [133]. These methodologies enable the simultaneous detection of multiple glucosinolates, thus streamlining the analytical process.

Furthermore, the integration of MS with chromatographic techniques has significantly enhanced glucosinolate detection. Wu et al. utilized LC-MS/MS in multiple reaction monitoring mode to analyze glucosinolate profiles in red cabbage, demonstrating the method’s sensitivity and its capacity to rapidly provide detailed compositional data [33]. This combination improves detection limits and facilitates the identification of various glucosinolates within complex matrices, which is crucial for comprehensive profiling in high-throughput applications. Automation plays a pivotal role in these advancements, facilitating the rapid processing of samples with minimal human intervention. Techniques such as automated sample preparation and robotic liquid handling systems can significantly reduce analysis time and variability in results. However, the reference by Li et al. does not specifically address glucosinolate analysis; rather, it focuses on genetically modified organisms [134]. Consequently, it is inappropriate to support claims regarding automation in glucosinolate detection based on this source. Furthermore, the proposed application of biosensors and microfluidic devices for real-time monitoring and analysis of glucosinolates lacks direct corroboration from the cited references [135,136]. In conclusion, the miniaturization and automation of glucosinolate detection methods are revolutionizing analytical practices by facilitating high-throughput analysis, minimizing resource consumption, and expediting analysis times. The integration of advanced chromatographic techniques with MS, alongside automation, is paving the way for more efficient and effective glucosinolate profiling.

### 5.3. Application of Machine Learning (ML) and Artificial Intelligence

ML and artificial intelligence algorithms can be employed to optimize detection methods, predict glucosinolate content, and classify plant materials based on their glucosinolate profiles. The application of ML and artificial intelligence (AI) in refining detection techniques, forecasting glucosinolate levels, and categorizing plant materials according to their glucosinolate profiles represents an emerging area of research that holds significant promise for agricultural and food sciences. Glucosinolates, a group of sulfur-containing compounds primarily located in Brassica species, are acknowledged for their health-promoting properties, which include cancer prevention and antioxidant effects [99,127,137]. The integration of ML and AI has the potential to enhance the efficiency and accuracy of glucosinolate analysis, which is critical for both breeding programs and food quality assessment.

One of the key applications of machine learning in this context is the enhancement of detection methods. Traditional techniques for glucosinolate quantification, such as HPLC, while effective, can be time-consuming and necessitate extensive sample preparation [92]. Recent advancements in ML algorithms have demonstrated their ability to analyze complex datasets generated from chromatographic techniques, enabling rapid identification and quantification of glucosinolates [23]. For instance, the GLS-Finder platform employs UPLC coupled with HRMS to conduct qualitative and semi-quantitative analyses of glucosinolates, significantly reducing analysis time [23]. Furthermore, ML can be trained to predict glucosinolate profiles based on various growth conditions and environmental factors, thereby facilitating more targeted breeding strategies [127,138]. In addition to optimizing detection methods, ML and artificial intelligence (AI) can be utilized to predict glucosinolate content in various plant materials. Factors such as soil composition, climatic conditions, and plant genetics significantly influence glucosinolate levels [138]. Statistical modeling approaches, including regression analysis and neural networks, have been effectively employed to forecast glucosinolate concentrations in crops such as Chinese cabbage and kale based on these variables [127,138]. For instance, studies have demonstrated that environmental factors like temperature and humidity are correlated with glucosinolate accumulation, facilitating predictive models that assist in selecting optimal growing conditions for desired glucosinolate profiles [131,138].

Furthermore, artificial intelligence (AI) can facilitate the classification of plant materials based on their glucosinolate content. By employing supervised learning techniques, researchers can categorize different cultivars of Brassica species according to their glucosinolate profiles, which is essential for both breeding and consumer preferences [137]. ML algorithms are capable of analyzing spectral data from methods such as hyperspectral imaging to classify plant materials rapidly and non-destructively, providing a valuable tool for quality control in agricultural practices [139]. This classification capability can also extend to identifying plant varieties with enhanced health-promoting properties, thereby guiding breeding programs aimed at improving nutritional quality [137]. Thus, the integration of ML and AI into glucosinolate research offers significant advancements in detection methods, predictive modeling, and the classification of plant materials. These technologies not only enhance the efficiency of glucosinolate analysis but also contribute to the development of crops with optimized health benefits, aligning with the increasing demand for functional foods in the market.

## Figures and Tables

**Table 1:** Common analytical techniques and extraction and purification procedures of glucosinolates.

Analytical Techniques	Extraction	Purification
HPLC	Solvent Extraction	Ion-Exchange Chromatography
LC-MS	UAE	Gel Permeation Chromatography (GPC)
GC-MS	MAE	DEAE-Sephadex and Sephadex G-10
NMR	Enzymatic Extraction Method	
NIRS	Cold Methanol Extraction	
ELASA		

Note: This table recapitulates the analytical techniques and extraction and purification procedures for glucosinolates in the recent decade, as referred to above.

**Table 2:** The individual compounds of glucosinolates found in cruciferous plants.

Family	Species	Tissues and Organs	GSLs Compounds
Brassicaceae	*Brassica napus*	Seed, leaves, stems, roots	6-15
	*Brassica juncea*		7-17
	*Capsella bursa-pastoris Medic.*		3-7
	*Brassica oleracea. var. botrytis*		4-9
	*Brassica rapa*		2-11
	*Brassica carinata A Braun*	Seed, leaves, stems	3-8
	*Camelina sativa*		3-12
	*Camelina rumelica subsp. rumelica*		
	*Camelina macrocarpa*		
	*Brassica oleracea var capitata*	Seeds, leaves	3-12
	*Brassica oleracea convar capitata var alba*	Florets, seedlings	3-14
	*Brassica oleracea var italica*	Seed, leaves, stems, roots, seedlings	7-16
	*Raphannus sativus*	Roots, seeds	6-14
	*Arabidopsis thaliana*	Leaf, florets, flowers, seedlings	3-23

Note: This table references some previous reports [2,111,112,113].

**Table 3:** The profiles of glucosinolates detected in cruciferous plants.

Glucosinolate Types	Chemical Names	Common Names	Characterization Methods
Aliphatic GSLs	Methyl GSL	Glucocapparin	M, N
	1-Methylethyl GSL	Glucputranjivin	U, I, M, N
	3-Methoxycarbonyl-propyl GSL	Glucoerypestrin	N
	Ethyl GSL	Glucolepidiin	Thiourea-type
	4-Oxoheptyl GSL	Glucocapanglin	Deducted from I and 5-oxooctanoic acid
	5-Oxoheptyl GSL	Gluconorcappasalin	Thiourea-type, I compared with GSL
	5-Oxooctyl GSL	Glucocappasalin	U, I of GSL Mn
	2-Hydroxy-2-methylpropyl GSL	Glucoconringiin	M, N
	(2S)-2-Hydroxy-2-methylbutyl GSL	Glucocleomin	N of desGSL
	(1R)-1-(Hydroxymethyl)-propyl GSL	Glucosisaustricin	M, N of desGSL
	(2S)-2-Methylbutyl GSL	Glucojiaputin	U, I, M, N of GSL and desGSL
	(1S)-1-Methylpropyl GSL	Glucocochlearin	M, N of GSL Mn
	3-(Methylsulfanyl)propyl GSL	Glucoibervirin	M, N of GSL
	4-Oxoheptyl GSL	Glucocapangulin	Deduction from I,5-oxooctanoic acid
	4-(Methylsulfanyl)butyl GSL	Glucoerucin	U, I, M, N of GSL
	5-(Methylsulfanyl)pentyl GSL	Glucoberteroin	U, I, M, N of GSL; U, M, N of desGSL
	6-(Methylsulfanyl)hexyl GSL	Glucolesquerellin	U, I, M, N of GSL
	(R)-11-(Methylsulfinyl)-propyl glucosinolate	Glucoiberin	M, N, X-ray of GSL; U
	(R/S)-4-(Methylsulfinyl)-butyl glucosinolate	Glucoraphanin	M, N of GSL, U
	(R/S)-5-(Methylsulfinyl)pentyl GSL	Glucoalyssin	M, N of GSL
	(R/S)-6-(Methylsulfinyl)-hexyl GSL	Glucohesperin	U, I, M, N of GSL
	(R/S)-8-(Methylsulfinyl)-octyl GSL	Glucohirsutin	U, I, M, N of GSL
	(R/S)-9-(Methylsulfinyl)-nonyl GSL	Glucoarabin	U, I, M, N of GSL
	(R/S)-10-(Methylsulfinyl)decyl GSL	Glucocamelinin	M, N of GSL
	3-(Methylsulfonyl)-propyl GSL	Glucocheirolin	M of GSL; N of desGSL
	4-(Methylsulfonyl)butyl GSL	Glucoerysolin	M of GSL; M, N of desGSL
	(R/S, 3E)-4-(Methylsulfiny1)-but-3-enyl GSL	Glucoraphenin	M, N of GSL; U, N of desGSL
	(R)-4-(Cystein-S-yl) butyl GSL	Glucorucolamine	M, N of desGSL
	4-(β-D-Glucopyranosyl-disulfanyl)-butyl GSL	Diglucothiobeinin	M of GSL; M, N of desGSL
	6-Benzoyl-4 (methylsulfanyl)butyl GSL	6′-Benzoyl-glucoerucin	U, M, N of desGSL
	6′-Benzoyl-4(methylsulfinyl)butyl GSL	6′-Benzoyl-glucopharanin	U, M, N of desGSL
	(R/S,3E)-6-Sinapoyl-4 (methylsulfinyl)but-3-enyl GSL	6′-Sinapoyl-glucoraphenin	U, I, M, N of desGSL
	Allyl glucosinolate	Sinigrin	M, N, X-ray of GSL; U
	But-3-enyl GSL	Gluconapin	M, N of GSL; U,
	Pent-4-enyl GSL	Glucobrassicanapin	M of GSL
	(2S)-2-Hydroxypent 4-enylGSL	Gluconapoleiferin	M of GSL
	(2R)-2-Hydroxybut 3-enylGSL	Progoitrin	M, N of GSL; U, M, N of desGSL
	(2S)-2-Hydroxybut 3-enylGSL	Epiprogoitrin	M, N of GSL; U, M, N of desGSL
	Chemical Names	Common Names	Characterization Methods
Glucosinolate types	(1R)-2-Bezoyloxt-1-methylethyl GSL	Glucobenzosisymbrin	U, I of ITC
Aromatic GSLs	(1R)-1-(Benzoyloxymethyl) propyl GSL	Glucobenzsisaustricin	Thiourease-type, I compared with GSL
	Benzyl GSL	Glucotropaeolin	M, N of GSL; U, M, N of desGSL
	3-Hydroxybenzyl GSL	Glucolepigramin	M of GSL; M, N of desGSL
	3-Methoxybenzyl GSL	Glucolimnanthin	M, N of GSL; U, M, N of desGSL
	4-Hydroxybenzyl GSL	Glucosinalbin	U, M, N of GSL Mn
	4-Methoxybenzyl GSL	Glucoaubrietin	M, N of desGSL
	3,4-Dihydroxybenzyl GSL	Glucomatronalin	M of GSL
	4-Hydorxy3-methoxybenzyl GSL	3-Methoxysinalbin	U, M, N of desGSL
	3-Hydroxy-4-methoxybenzyl GSL	Glucobretschneiderin	U, I, M, N of GSL
	4-Hydorxy-3,5-dimethoxybenzyl GSL	3,5-Dimethoxy-sinalbin	U, M, N of desGSL
	2-Phenylethyl GSL	Gluconasturtiin	N of GSL; U, M, N of desGSL
	2-hydroxy-2-phenylethyl GSL	Glucobarbarin	M, N of GSL Mn
	(2R)-2-Hydroxy-2-phenylethyl GSL	Epiglucobarbarin	M, N of GSL Mn
	2-(4-Methoxy-phenyl) ethyl GSL	Glucoarmoracin	N of GSL, M, N of desGSL
	(2R)-2-Hydroxy-2-(4-hydroxyphenyl) ethyl GSL	p-Hydroxy-epiglucobarbarin	M, N of GSL; U, M, N of desGSL
	(2S)-2-Hydroxy-2(4-hydroxyphenyl) ethyl GSL	p-Hydroxy-glucobarbarin	U, M, N of desGSL
	4-(4′-O-acetyl-α-c-4-rhamnopyranosyloxy)-benzyl GSL	4-Mcetyl-glucomoringin	M of GSL and ITC
	6′-Isoferuloyl-2 phenylethyl GSL	6′-Isoferuloyl gluconasturtiin	M of GSL, U
	6′-Isoferuloyl-(2R) 2-hydroxy-2phenylethyl GSL	6′-Isoferuloyl epiglucobarbarin	M, N of GSL; U
	6′-Isoferuloyl-(2S) 2-hydroxy-2phenylethyl GSL	6′-Isoferuloyl glucobarbarin	M, N of GSL; U, M, N of desGSL
	4-(α-_L_-Rhamnopyranosyloxy) benzyl GSL	Glucomorinigin	M, N of GSL Mn
	4-Methoxyindol-3-yl GSL	Glucorapassicin M	U, I, M, N of synthesized GSL
Indolic GSLs	Indol-3-ymethyl GSL	Glucobrassicin	U, I, M, N of GSL Mn
	4-Hydroxyindol-3-ylmethyl GSL	4-Hydroxy-glucobrassicin	M of GSL; U, M, N of desGSL
	4-Methoxyindol-3-ylmethyl GSL	4-Methoxy-glucobrassicin	U, M, M, N of GSL Mn
	1-Methoxyindol-3-ylmethyl GSL	Neoglucobrassicin	U, I M, N of GSL; M, N of desGSL
	1,4-Dimethoxyindol-3-ymethyl GSL	1,4-Dimethoxy-glucobrassicin	U, M, N of desGSL
	1-Acetylindol-3-ymethyl GSL	N-Acetyl-glucobrassicin	M of desGSL
	1-Sulfoindol-3-ylmethyl GSL	N-Sulfo-glucobrassicin	U, I, M, N of GSL
	6′-Isoferuloylindol-3-ylmethyl GSL	6′-Isoferuloyl-glucobrassicin	M of GSL; U, M, N of desGSL

Note: This table references some previous reports [2,114,115]. MS stands for M, NMR stands for N, UV stands for U, IR stands for I, and desGSL stands for Mn.

## Data Availability

No new data were created or analyzed in this study. Data sharing is not applicable to this article.
